# Mouse Gambling Task reveals differential effects of acute sleep debt on decision-making and associated neurochemical changes

**DOI:** 10.1093/sleep/zsy168

**Published:** 2018-10-10

**Authors:** Elsa Pittaras, Jacques Callebert, Rodolphe Dorey, Mounir Chennaoui, Sylvie Granon, Arnaud Rabat

**Affiliations:** 1 Unité Fatigue et Vigilance, Département Environnements Opérationnels, Institut de Recherche Biomédicale des armées, Brétigny-sur-Orge cedex, France; 2 Equipe ‘Neurobiologie de la prise de décision’, Neuro-PSI, CNRS UMR 9197, Orsay, France; 3 Equipe d’accueil VIgilance FAtigue et SOMmeil (VIFASOM) EA 7330 - Université Paris 5 Descartes, Paris, France; 4 Biology Department, Stanford University, Stanford, CA; 5 Service de Biochimie et Biologie Moléculaire, Hôpital Lariboisière, Paris, France

**Keywords:** sleep, decision-making, interindividual differences, brain, monoamines, dorsal striatum, frontal cortex, sleep deprivation

## Abstract

Sleep loss is associated with sleepiness, sustained attention, and memory deficits. However, vulnerability of higher cognitive processes (i.e. decision making) to sleep debt is less understood. Therefore, a major challenge is to understand why and how higher cognitive processes are affected by sleep debt. We had established in mice correlations between individual decision-making strategies, prefrontal activity, and regional monoaminergic levels. Now, we show that acute sleep debt (ASD) disturbs decision-making processes and provokes brain regional modifications of serotonin and dopamine that could explain why ASD promotes inflexible and more risk-prone behaviors. Finally, we highlight, for the first time, that in a large group of healthy inbred mice some of them are more sensitive to ASD by showing inflexible behavior and decision-making deficits. We were also able to predict mice that would be the most vulnerable to ASD depending of their behavior before ASD exposure.

**Statement of Significance**

Using a decision-making task under ambiguity in mice completed by biochemical and neuroanatomical approaches, we characterized markers of decision-making deficits and interindividual differences regarding cognitive deficits after acute sleep debt (ASD). We highlight for the first time that in a large group of healthy inbred mice, 21% are more sensitive to sleep debt than others, showing extreme inappropriate decision-making profiles and inflexible behaviors. We were also able to predict which mice would be the most vulnerable to ASD. We feel that these exciting novel findings open new avenues and come amidst substantial efforts within the scientific community for understanding neurobiological substrates leading to individual vulnerability to ASD that is at present a matter of major interest for our society.

## Introduction

In everyday life, we have to make frequent choices. When situations are complex, ambiguous, or novel, higher executive functions become necessary, i.e. planning, reasoning, problem solving, decision making [1]. These executive functions rely on other executive processes such as behavioural inhibition, working memory, cognitive flexibility [1, 2], which require optimal functioning of parieto-frontal and fronto-striatal networks together with appropriate monoaminergic neurotransmissions [3]. Many factors such as cognitive load, anxiety, or stress are deleterious to these processes [1, 4]. Acute sleep debt (ASD) is one of these factors known to reduce metabolism in brain networks [5] leading, in addition to sleepiness, to cognitive deficits in humans [6]. More precisely, ASD is responsible both for sustained attention and working memory deficits and more recently for cognitive rigidity through perseverative errors [7–10].

Neurobiological hypotheses explaining cognitive deficits related to ASD are not well understood. On one hand, the brain serotonergic system is involved in decision-making [11] and prefrontal serotonin (5-HT) depletion in marmoset is sufficient to produce cognitive inflexibility [12]. The 5-HT neuromodulator system is also implicated in sleep regulation [13] and is modified by sleep loss [14]. On the other hand, ASD is responsible for higher dopamine (DA) levels in striatum and thalamus [15]. ASD is also associated with riskier decision-making to optimize gains on gambling tasks [16–18] through an increase preference for rewarding stimuli [18, 19] and with addictive behavior [20, 21]. Overall previous studies highlight the importance of monoamines for decision-making disorders related to ASD but with no clear evidence either for the executive processes engaged or for neural systems involved. We thus hypothesize here that ASD will modify monoamines levels in brain areas involved in decision-making processes and therefore will alter such higher cognitive processes.

Individual vulnerability to sleep debt on sustained attention and working memory processes have been shown [22–26]. These individual differences already exist at baseline and ASD amplifies them [27, 28]. However, interindividual vulnerability of decision-making processes related to sleep loss is largely unknown. Most studies in humans and rodents show global performance deterioration despite well-known interindividual differences regarding sleep lost vulnerability.

Major challenges are to understand neurobiological systems involved in decision-making that are impaired by ASD and the reasons for individual differences in vulnerability to ASD of such higher executive functions. Animal models are an interesting way to ask such questions [29]. We developed a decision-making task in mice: the Mouse Gambling Task (MGT) [30, 31], inspired by the Iowa Gambling Task [32]. This task requires mice to gamble for food by making choices in a four-arm maze between more or less advantageous options in the long term with uncertain and probabilistic reward delivery [30, 31]. Our MGT was set up in two virtual phases: an exploration phase during which mice explored the different options and, an exploitation phase during which mice developed preferences evaluating the value of each option regarding their own preferences [30–33]. Therefore, the MGT allows us to study the effect of ASD at two different steps of the decision-making process states and we thus hypothesize that decision-making will be more altered if ASD is applied before the settle of mice strategies. MGT also allows the dissociation between three main profiles of decision-making strategies: significant preference for long-term advantageous choices but also showing rigid behavior, preference for exploratory behavior that leads to risk-taking and an intermediate mice strategy consisting of maintaining exploration of multiple options with moderate risk [30, 31]. We also established that these individual profiles rely on specific monoaminergic levels in the prefrontal cortex, the striatum, and the hippocampus. Therefore, by using MGT we can study decision-making deficits related to ASD, associated monoaminergic activity and we can question whether ASD differentially impacts individual decision-making profiles in mice and if it is predictable.

We show that ASD increased impulsivity, altered decision-making strategy, DA and 5-HT level in brain areas involved in such higher cognitive processes. We also show for the first time that ASD applied before the establishment of mice preferences during a decision-making task had a more important effect on 21% of a large group of healthy mice and we were able to predict this vulnerability to ASD for 83% of them (18% of the total group of mice).

## Materials and Methods

### Animals

The study used 139 male C57Bl/6J mice between 3 and 6 months old at the beginning of the experiments (Charles’ River, Orleans, France). Mice were housed by three or four, in a temperature controlled room (21°C ± 2°C), with a 12 light/12 dark cycle (lights on at 08:00 am). Experiments were performed between 09:00 am and 06:30 pm. For the sucrose preference task mice were temporally housed individually for 3 weeks. During the MGT, mice were food deprived (individually maintained at 85% of the free feeding weight). Water was always provided *ad libitum*. For experiments in which an ASD was applied between the fifth and the sixth days we used 23 ASD mice and 23 control mice. For experiments in which ASD was applied between the second and the third days we used 28 ASD mice and 13 control mice added to the 23 control mice of the first group. For control experiments (corticosterone dosage, locomotor activity recording, anxiety scoring, and sucrose preference measurement), 52 additional mice were used.

Animals were treated according to the ethical standards defined by the “Centre National de la Recherche Scientifique” for animal health and care with strict compliance with the EEC recommendations (n°86/609). Interindividual studies need a large number of animals; however, the number of animals used was reduced to the minimum.

### Mouse Gambling Task

**Figure 1.**
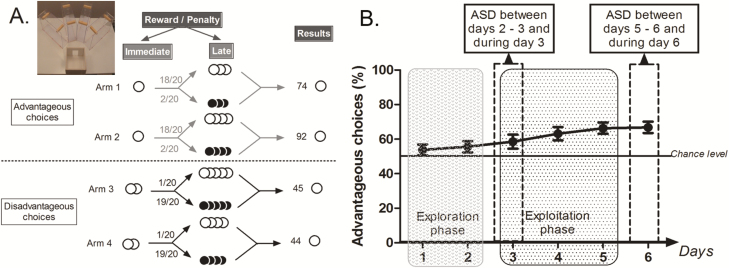
MGT and ASD protocol. A. Schematic representation of the MGT experimental design. White circles represent food pellets and black circles quinine pellets. Advantageous choices gave access to one food pellet and disadvantageous choices gave access to two food pellets. Then mouse can find three or four food pellets (18/20) or quinine pellets (2/20) in advantageous choices and four or five food pellets (1/20) or quinine pellets (19/20) in disadvantageous choices. If a mouse chooses only the arm 1 during 20 trials, it will obtain 74 pellets (1 × 20 immediate food pellets reward and 18 × 3 food pellets as late reward). Likewise, if a mouse chooses only the arm 2, 3, or 4 it will obtain respectively 92, 45, or 45 food pellets. We distinguished advantageous choices from disadvantageous ones because mice earned more pellets after 20 trials by choosing the advantageous ones. B. Schematic representation of the mean of the percentage of advantageous choices of 23 mice during 6 days of the MGT with illustration of the exploration phase, during which mice explore options, and then an exploitation phase during which mice exploit their knowledge of the value of each option. During the exploration phase, mouse preferences are close to chance level while during the exploitation phase mice progressively prefer advantageous options. One group of mice was sleep deprived between the second and the third day (before the exploitation phase) and another group of mice was sleep deprived between the fifth and the sixth day (after the exploitation phase).

Experiments were conducted as previously described [31]. The task took place in a maze with four transparent arms (20 cm long × 10 cm wide) containing an opaque start box (20 cm × 20 cm) and a choice area (Figure 1A). We used standard food pellets as a reward (dustless Precision Pellets, Grain-based, 20 mg, BioServ, New Jersey) and food pellets previously steeped in a 180-mM solution of quinine as penalty. The quinine pellets were unpalatable but not inedible.

There were four different arms: two gave access to “advantageous” choices in the long term and two others gave access to “disadvantageous” choices in the long term. In “advantageous” arms mice systematically found 1 pellet (“small reward”) before a cup containing food pellets on 18 trials out of 20 and quinine pellets for two remaining trials (Figure 1A). In the “disadvantageous” arms mice found two food pellets (“large reward”) before a cup containing quinine pellets on 19 trials out of 20 and food pellets in the remaining trial (Figure 1A). Advantageous choices are at first less attractive than disadvantageous choices because of the small immediate reward (1 pellet *vs.* 2 pellets). Despite this apparent lower attractiveness advantageous choices are advantageous in the long term because food pellets had higher probability of being found than quinine pellets. Conversely disadvantageous choices are less advantageous in the long term because animals had a higher probability of finding quinine pellets than food pellets. Therefore, mice had to favor the small immediate reward (advantageous choices) to obtain the highest amount of pellets as possible at the end of the day [30, 31].

Each animal realized 20 trials by day: 10 trials in the morning (between 09:00 am and 01:00 pm) and 10 trials during the afternoon (between 02:00 pm and 06:00 pm). We scored the percentage of advantageous choices by day [(number of advantageous choices / number of total choices) × 100], the food pellet consumption (pellets earned), the number of quinine pellets obtained (but not eaten) and the latency to choose the arm during trials. We also calculated a rigidity score by measuring how many times the mouse chose the same arm. For example, a rigidity score of 25% means that the mouse chose arm by chance and a rigidity score of 100% that the mouse chose always the same arm. Therefore, a rigidity score of 50% reflected that the mouse had chosen one arm twice as much as the others, and a rigidity score of 75% that animal had chosen one arm three times more often than the others [31].

### Acute sleep debt

#### Apparatus

Animals were sleep deprived by being placed in a transparent cylinder (PVC, 45 cm height) connected to a shaking platform (diameter 30 cm; Viewpoint) [34]. Sleep was prevented by the platform bouncing randomly with variable frequency (stimulation every 100–200 ms), intensity and duration (from 20 to 40 ms). Number (from 2 to 4) and duration of stimulations (from 10 to 30 ms of interval) were also randomly determined. This method has been shown to successfully decrease slow wave sleep (also named NREM sleep) (from 35.8 ± 1.4% to 9.2 ± 2.7%, *p* < 0.001) and paradoxical sleep (also named REM sleep) (from 9.5 ± 2.4% to 0.03 ± 0.01%, *p* < 0.05) during sleep debt. This method was also followed by both slow wave and paradoxical sleep rebound (respectively from 33.5 ± 2.6% to 58.2 ± 5.9%, *p* < 0.05 for slow wave sleep and from 4.6 ± 1.3% to 16.1 ± 2.1%, *p* < 0.05 for paradoxical sleep) [34]. Therefore, it is more accurate to use the term “Acute Sleep Debt (=ASD)” instead of total sleep deprivation even if mice could only sleep during 9.2% of the 23 hours (= 2 hours). The platform was partitioned into four identical compartments (12 cm^2^; one mouse in each compartment) with food and water available *ad libitum*. Software controlled the shaking parameters (Viewpoint, France) during the 23 hours of ASD.

#### Protocol

One group of mice was sleep deprived after the exploitation phase (between day 5 and 6 and during day 6), while another group of mice was sleep deprived before the exploitation phase (between day 2 and 3 and during day 3, Figure 1B) [31]. For the first group, 23 mice were put in the shaking apparatus to induce ASD for 23 hours from the end of the fifth day until the end of the sixth day of MGT (from 05:00 pm on the fifth day to 04:00 pm on the sixth day). For the second group, 28 mice were put in the shaking apparatus to induce ASD for 23 hours from the end of the second day until the end of the third day of MGT (from 05:00 pm on the second day to 04:00 pm of the third day). Control mice were put into cylinders without shaking. One MGT session occurred in the morning and one in the afternoon. Animals were taken out of cylinders for morning and afternoon MGT task and were then put back in cylinders between these two sessions. Finally, all mice went back to their home cages exactly 23 hours after the beginning of ASD, i.e. after the afternoon session.

### Control experiments

#### Corticosterone dosage

Mice were sacrificed by decapitation and blood samples were collected in plastic tubes containing appropriate amounts of anticoagulants (EDTA: 0.1 mL of 10% disodium EDTA solution for 2 mL of blood). After 10 minutes of centrifugation at 10000 g, plasma samples were collected into plain tubes, containing 10% EDTA and stored at −80°C. Plasma corticosterone was measured by an enzyme immunoassay commercial kit (Correlate-EIA; Assay Designs, Ann Arbor, MI). The sample preparation was adapted from the corticosterone guidebook with a final plasma dilution of 1/50. The sensitivity of this assay is higher (18.6 pg/mL) than the RIA (5 ng/mL) method [35]. Corticosterone levels were determined after 23 hours of ASD with the cylinder activated (ASD mice), or not activated (control mice) or in mouse home cages (home cage control).

#### Locomotor activity

Locomotor activity was analyzed by putting animals in transparent chambers (21 cm × 13 cm) for 30 minutes, i.e. the approximate duration of MGT. Experiment was recorded on camera and analyzed off-line with ANY-Maze Software. ANY-Maze Software tracked the mouse to measure the distance traveled during 30 minutes’ task immediately after 23 hours of ASD (ASD mice, with the cylinder activated, *n* = 8; control mice, with the cylinder not activated, *n* = 8) around 02:00 pm.

#### Sucrose preference task

The sucrose preference task measured animal’s sensitivity to reward after ASD [36]. Animals were isolated in home cages 2 weeks before the experiment and during the experiment with two glasses of water bottles. They were then habituated to drink only sucrose solution (1%) for 24 hours in their home cage. Animals were thereafter put in the sleep deprivation apparatus for 23 hours (ASD mice, with the cylinder activated, *n* = 8; control mice, with the cylinder not activated, *n* = 8). Sucrose preference score was measured after 23 hours of ASD for each mouse by putting two glass bottles in their home cage, one with water and the other one with sucrose solution. The sucrose preference score was calculated by: [(mL of sucrose solution drunk) / (mL of sucrose solution and water drunk)] × 100.

#### Elevated Plus Maze

The Elevated Plus Maze consists of two open arms (30 × 5 cm) and two wall-enclosed arms (30 × 5 × 25 cm) connected by a central platform (5 × 5 cm) [37]. Light intensity on the open arms was adjusted to 120 lux. The apparatus was elevated 75 cm above the floor. Behavioral testing started by placing a mouse in the central area; facing a closed and an open arm. Exploratory behavior was monitored by a video motility system (Video track, Viewpoint, France); over a 5-minute period, quantified and stored on the computer. The behavioral parameters were the percentage of time spent in open arms after 23 hours in the ASD cylinders activated (ASD mice, *n* = 8) or not (control mice, *n* = 8). Visit of an open arm was scored as soon as the mouse placed its two forepaws in the arm.

### Rate of monoamines

Experiments were conducted as previously described [30, 31].

#### Brain extraction

Brains were removed at the end of the last day of the MGT. Animals were slightly anesthetized with Isoflurane (Iso-Vet, 1000 mg/g) before cervical dislocation. Brains were rapidly removed and stored at −80°C.

#### Brain section and punch

Brains were placed at −20°C the day before slicing. One hour before slicing, brains were brought to the cryostat and maintained at −13°C. Coronal sections (140 μm) were taken and punches (diameter: 0.75 mm) of each brain region were precisely localized using the mouse atlas [38].

We punched six regions of interest from both hemispheres: orbitofrontal cortex (lateral, median, dorsolateral, and ventral, OFC), Pre and infra limbic cortex (PrL and IL), insular cortex (CIns) (agranular and granular insular cortex, dorsal, and ventral), the amygdala (basolateral amygdala and amygdalian nucleus, Amy), the hippocampus (H), and caudate putamen (CPu).

#### HPLC dosage

Amount of DA, 5-HT, 5-hydroxyindoleacetic acid (5-HIAA), and noradrenaline (NA) were quantified by using high-performance liquid chromatography (HPLC). DA turnover (3,4-dihydroxyphenylacetic acid—DOPAC, homovanillic acid—HVA) was measured only in the CPu because of the high level of DA in that region.

Prior to analysis, brain tissues were crushed in 350 μL of 0.2 M perchloric acid and centrifuged at 22000 g for 20 minutes at 4°C. The supernatants were collected and filtered through a 10-kDa membrane (Nanosep, Pall) by centrifugation at 7000 g. Then, a 20-μL aliquot of each sample was analyzed for 5-HT by fluorometric detection [39, 40]. The amounts of catecholamines (DA and norepinephrine) were measured by electrochemical detection on a serial array of coulometric flow-through graphite electrodes (CoulArray, ESA, 39). Analyses, data reduction, and peak identification were fully automated. Results were expressed as femtomoles/mg of fresh tissue [39, 40].

### Statistical analyses

#### Subgroup formation

To distribute animals among groups according to their MGT preference we calculated for each animal the mean of the percentage of advantageous choices for the 30 last trials, i.e. when preference was stable and used a *k*-mean clustering analysis with Statistica software (version 12) [31]. Each animal belonged to a set that had the mean closest to its own preference value. Animals were separated into three subgroups: animals that chose a majority of advantageous options at the end of the experiment, called “safe”; animals that explored options until the end of the experiment, called “risky”; and animals that maintained some exploration of available options but favored advantageous options, called “average” [31].

#### Statistical analysis

Statistical analyses were performed using Statview software. For the data that showed normal distribution (Shapiro–Wilkinson test) and passed equal variance tests (*F* test), statistical analyses were performed using *t*-test. When data did not show normal distribution or pass equal variance tests, statistical analyses were performed using ranked signs Wilcoxon task, Kruskal–Wallis, or Mann–Whitney U-tests. Bonferroni correction was applied for multiple comparisons. Results were reported as means ± SEM. *P* values ≤ 0.05 were considered statistically significant except when Bonferroni correction was necessary.

#### Linear regression

The linear regression analysis was done using R software to study the interaction between ASD, mean of mice preference during the exploration phase (day 1 and day 2) and time on mice’s preference during the MGT.

The formula was as below:

$$\begin{array}{l} {\text{Y}}_{}=\beta_{0}+\beta_{\text{1}}*\text{ASD }+\beta_{\text{2}}*\text{Post }+\beta_{\text{3}}*\text{Vulnerable }+\beta_{\text{4}}*\text{ASD}*\text{Post }+\beta_{\text{5}}*\text{Post}*\text{Vulnerable }+\beta_{\text{6}}*\text{ASD}*\text{Vulnerable }\\ +\beta_{\text{7}}*\text{ASD}*\text{Post}*\text{Vulnerable }+\varepsilon \end{array}$$

*Y* preferences or Rigidity;

ASD = {0,1}, defined as ASD = 0 if ASD was not applied for this mouse and ASD = 1 if ASD was applied for this mouse;

Post = {0,1}, defined as Post = 0 before ASD and Post = 1 after ASD;

Vulnerable = {0,1}, defined as Vulnerable = 0 if the mean of day 1 and day 2 preferences for a mouse was above 40% of advantageous choices and Vulnerable = 1 if the mean of day 1 and day 2 preferences for a mouse was below 40% of advantageous choices;

*ε* as the error.

*H*_0_ was defined as “there is no effect of ASD.” Therefore, if *P* values ≤ 0.05, *H*_0_ is rejected and we concluded that we have an effect of ASD.

## Results

### Main effect of ASD applied after exploitation phase

**Figure 2.**
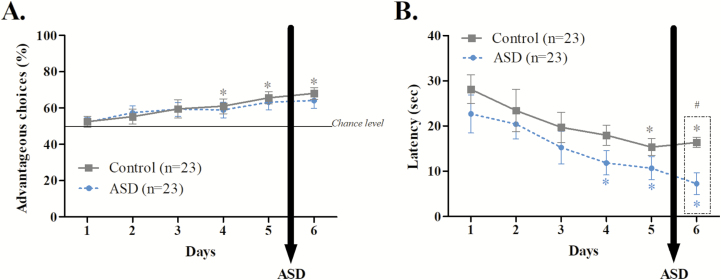
Effects of ASD applied after the exploitation phase. A. Percentage of “advantageous” choice (mean ± SEM) for control (full line, *n* = 23) and ASD mice (dotted blue line, *n* = 23). Only control mice showed an increase of advantageous preferences compared with day 1 (*p < 0.01). No difference existed between control and ASD mice (*p* > 0.05). B. Mean ± SEM of response latencies for each day for control (full line, *n* = 23) and ASD mice (dotted blue line, *n* = 23). # represented significant differences between control and ASD mice (^#^*p* < 0.05). Control and ASD mice showed a decrease of response latency compared with day 1 (*p < 0.01).

As illustrated in Figure 2A, preferences for advantageous choices of ASD mice did not differ from those of control mice when ASD is applied after the exploitation phase of the MGT (day 1 *U* = 244.000, *p* = 0.65; day 2 *t* = −0.369, *p* = 0.71; day 3 *t* = −0.034, *p* = 0.97; day 4 *U* = 261.500, *p* = 0.95; day 5 *t* = −0.420, *p* = 0.65; day 6 *U* = 231.500, *p* = 0.47). Mice preferences for advantageous options increased over time only for the control group (Bonferroni correction, *p* < 0.01; control mice: day 1 *vs*. day 2 *Z* = −0.763, *p* = 0.44; day 1 *vs*. day 3 *Z* = −1.217, *p* = 0.22; day 1 *vs*. day 4 *Z* = −2.483, *p* = 0.01; day 1 *vs*. day 5 *Z* = −3.397, *p* = 0.0007; day 1 *vs*. day 6 *Z* = −3.734, *p* = 0.0002). We observed that mice preference for advantageous choices had only a tendency to increase for ASD mice after day 4 (Bonferroni correction, *p* < 0.01; ASD mice: day 1 *vs*. day 2 *t* = −1.973, *p* = 0.06; day 1 *vs*. day 3 *t* = −1.665, *p* = 0.11; day 1 *vs*. day 4 *Z* = −1.634, *p* = 0.1023; day 1 *vs*. day 5 *t* = −2.106, *p* = 0.04; day 1 *vs*. day 6 *Z* = −2.207, *p* = 0.02).

Mice did not differ from each other regarding their response latency except after ASD. Indeed, as shown in Figure 2B, both groups showed a decrease of their latency to choose after day 5 (Bonferroni correction, *p* < 0.01; control mice: day 1 *vs*. day 2, *Z* = −1.430, *p* = 0.15; day 1 *vs*. day 3, *Z* = −1.217, *p* = 0.22; day 1 *vs*. day 4, *Z* = −1.656, *p* = 0.09; day 1 *vs*. day 5, *Z* = −3.159, *p* = 0.001; day 1 *vs*. day 6, *Z* = −3.215, *p* = 0.001; ASD mice: day 1 *vs*. day 2, *Z* = −2.175, *p* = 0.02; day 1 *vs*. day 3, *Z* = −2.859, *p* = 0.04; day 1 *vs*. day 4, *Z* = −2.859, *p* = 0.004; day 1 *vs*. day 5, *Z* = −3.057, *p* = 0.002; day 1 *vs*. day 6, *Z* = −3.619, *p* = 0.0003), but ASD mice exhibited a response latency shorter than those of control mice on the sixth day (day 1 *U* = 217.000, *p* = 0.23; day 2 *U* = 190.000, *p* = 0.1; day 3 *U* = 220.500, *p* = 0.3; day 4 *U* = 178.000, *p* = 0.06; day 5 *U* = 202.000, *p* = 0.17; day 6 *U* = 121.000, *p* = 0.001).

In summary, ASD applied after the exploitation phase decreased mice response latency and had a tendency to affect their preferences for advantageous choices.

### Main effect of ASD applied before exploitation phase

**Figure 3.**
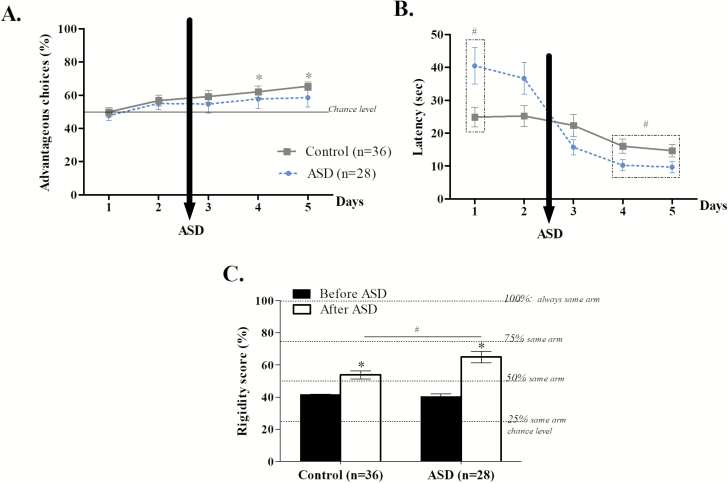
Effects of ASD applied before the exploitation phase. A. Mouse’ preference during the MGT represented as the percentage of advantageous choices (mean ± SEM) for control (full line, *n* = 36), and ASD mice (dotted line, *n* = 28). Control but not ASD mice preference differed from day 1 from the fourth day until the end (*p < 0.012). B. Means ± SEM of response latencies during each day for control (full line, *n* = 36) and ASD mice (dotted line, *n* = 28). # represents a significant difference between control and ASD mice (*p* < 0.05). C. Rigidity scores were reflected by the percentage of chosen arms during the first 2 days and the last 2 days of the task, i.e. before and after ASD. If the score is at 25%, animal, choose the four arms equally, while 100% reflected the choice of always the same arm. Rigidity scores during the first 2 (black) and last 2 (white) days for control and ASD mice. * represented differences between the beginning and the end of the task for one group of mice (*p* < 0.05). # represented differences between control and ASD mice (*p* < 0.05).

As shown in Figure 3A, only control mice developed a preference for advantageous options over time (Bonferroni correction, *p* < 0.012; control mice: day 1 *vs.* day 2, *U* = −2.117, *p* = 0.04; day 1 *vs.* day 3, *U* = −2.231, *p* = 0.02; day 1 *vs.* day 4, *U* = −2.269, *p* = 0.008; day 1 *vs.* day 5, *U* = −4.333, *p* < 0.001; ASD mice: day 1 *vs.* day 2, *t* = −2.230, *p* = 0.03; day 1 *vs.* day 3, *U* = −1.538, *p* = 0.12; day 1 *vs.* day 4, *U* = −2.066, *p* = 0.04; day 1 *vs.* day 5, *U* = −2.083, *p* = 0.03). However, preference for advantageous choices of ASD mice (ASD applied before the exploitation phase) did not differ from those of control mice (day 1 *U* = 424.000, *p* = 0.40; day 2 *t* = 0.350, *p* = 0.7272; day 3 *U* = 475.000, *p* = 0.69; day 4 *U* = 224.000, *p* = 0.34; day 5 *U* = 474.000, *p* = 0.68).

Even if ASD mice took more time to respond during the first day of the MGT (day 1 *U* = 340.500, *p* = 0.027), they significantly shifted just after ASD to very short response latency compared to control mice (day 3 *U* = 432.000, *p* = 0.33; day 4 *U* = 349.500, *p* = 0.04; day 5 *U* = 345.500, *p* = 0.03; Figure 3B).

We also evaluated animal’s rigidity before and after ASD. During the exploration phase, all mice chose around 40% of time the same arm (*U* = 483.500, *p* = 0.78). During the exploitation phase, preferences for advantageous arms emerged leading both control (*Z* = −3.725, *p* = 0.0002) and ASD mice (*Z* = −4.361, *p* < 0.0001) to choose more often the same arm. The rigidity score was increased after ASD (*t* = 2.560, *p* = 0.01), as ASD mice chose approximately 65 ± 3.1% of the time the same arm and control mice approximately 56 ± 2.9% of the time the same arm (Figure 3C).

In summary, ASD applied before the exploitation phase led to a lack of choices evolution for advantageous choices as well as an increase of rigid behavior and response latency.

### Control experiments

**Figure 4.**
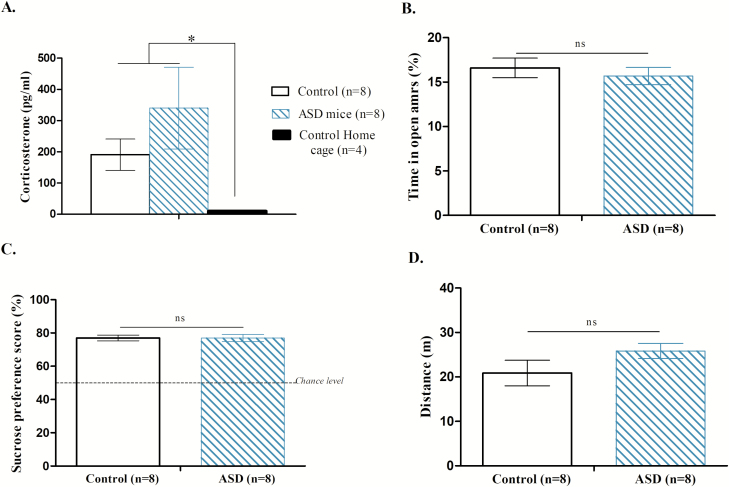
Control experiments. Effect of sleep debt (ASD) on stress, locomotion, sucrose preference, and anxiety. A. Level of corticosterone (pg/mL) for control mice (in the cylinder at rest, white bar, *n* = 8), ASD mice (*n* = 8) and control home cage mice (*n* = 4). * represents a significant difference between control home cage mice and ASD, control mice. B. Control (white bar, *n* = 8) and ASD (hatched bar, *n* = 8) mice did not differ from each other regarding the percentage of time spent in the open arms (mean ± SEM) during the Elevated Plus Maze (*p* > 0.5). C. Control (white bar, *n* = 8) and ASD (hatched bar *n* = 8) mice did not differ from each other regarding the sucrose preference score (mean ± SEM) after ASD (*p* > 0.5). D. Control (white bar, *n* = 8) and ASD mice (hatched bar, *n* = 8) did not differ from each other regarding the distance traveled in the locomotor activity paradigm (mean ± SEM [*p* > 0.5]).

**Figure 5.**
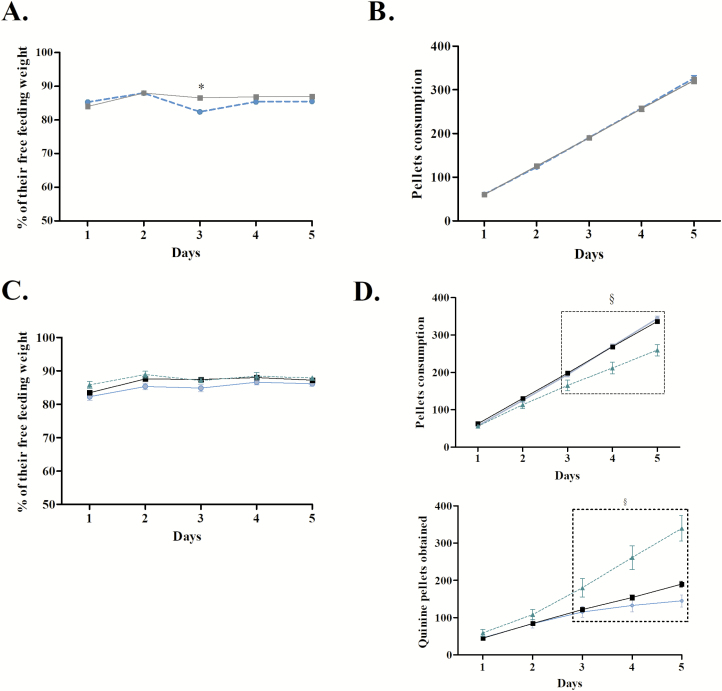
Mouse’s weight and pellets consumption during the MGT. A. Mouse’s weights during the MGT represented by the percentage of the free feeding weight (mean ± SEM) for control (full line, *n* = 36) and ASD mice (dotted line, *n* = 28). ASD mice lost more weight only the day after ASD (*p < 0.05). B. Pellet consumption during the MGT (mean ± SEM) for control (full line, *n* = 36) and ASD mice (dotted line, *n* = 28). Control and ASD mice ate the same amount of food pellets during MGT (*p* > 0.05). C. Weight changes during MGT in subgroups: safe (blue), average (black), and risky (green dotted line) ASD mice. Safe, average, and risky animals never differed from each other (*p* > 0.05). D. Food pellets and quinine pellets obtained by the three subgroups during the MGT. Risky animals obtained fewer food pellets and more quinine pellets than the other subgroups (^§^*p* < 0.05).

As shown in (Figure 4A and B), ASD neither produced an increase of corticosterone concentration (*U* = 23.500, *p* = 0.37) nor an increase of anxiety (*U* = 29.000, *p* = 0.75). ASD did not alter reward sensitivity (*U* = 0.014, *p* = 0.9891) nor locomotor activity (*U* = −1.797, *p* = 0.09) (Figure 4C and D). ASD led to weight loss only the day after ASD (Figure 5A, Bonferroni correction *p* < 0.01; day 1: *U* = 380.500, *p* = 0.09; day 2: *U* = 492.000, *p* = 0.87; day 3: *t* = 4.453, *p* = 0.0001; day 4: *U* = 349.000, *p* = 0.04; day 5: *t* = 2.002, *p* = 0.04), despite the fact that ASD and control mice consumed the same amount of food pellets (day 1: *U* = 435.000, *p* = 0.35; day 2: *U* = 489.500, *p* = 0.84; day 3: *U* = 493.500, *p* = 0.89; day 4: *t* = 1.427, *p* = 0.1586; day 5: *t* = 1.565, *p* = 0.1227; Figure 5B).

In summary, ASD had no effect on animal’s activity, stress, sensitivity to reward, pellet consumption or anxiety but led to a loss of weight just the day after ASD.

### Changes of brain monoamines amounts at the end of MGT when ASD was applied before the exploitation phase

**Figure 6.**
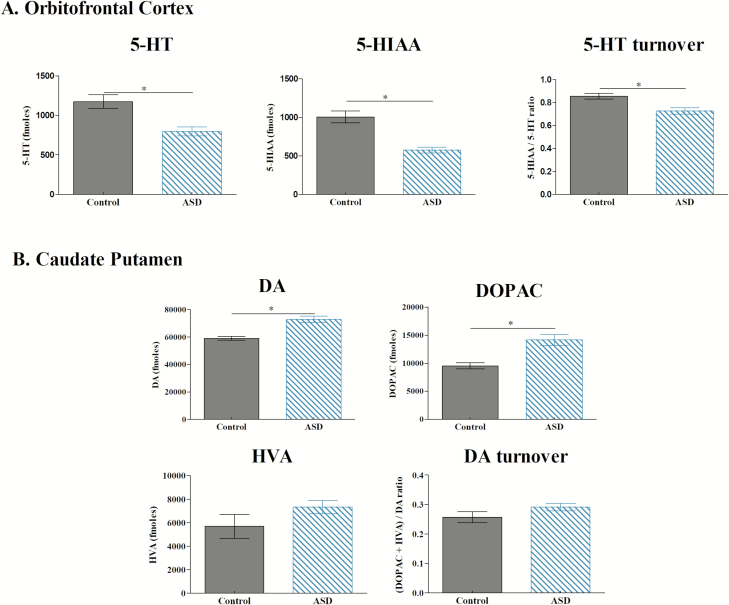
Quantification of serotonin and DA after the MGT. A. Quantification of serotonin (5-HT), 5-HIAA, and 5-HT turnover (5-HT/5-HIAA) in the OFC for control (*n* = 3) and ASD (*n* = 12) mice. ASD-mice exhibited lower 5-HT, 5-HIAA, and 5-HT turnover (*p < 0.05). B. DA, DOPAC, HVA amounts, and DA turnover (DA/DOPAC + HVA) in the CPu for control (*n* = 3) and ASD (*n* = 12) mice. ASD mice showed higher amounts of DA and DOPAC (*p < 0.05).

As shown in Figure 6A, ASD animals exhibited lower 5-HT levels (*U* = 1.000, *p* = 0.01), 5-HIAA level (*t* = 5.309, *p* = 0.001) and 5-HT turnover (*t* = 2.387, *p* = 0.03) in the OFC. Moreover, ASD induced higher 5-HT level in the CIns (*U* = 3.500, *p* = 0.04) but no change in 5-HIAA (*t* = −1.121, *p* = 0.28) or 5-HT turnover (*t* = 0.365, *p* = 0.72, Table 1). In the hippocampus, 5-HT levels were lower following ASD (*t* = 4.447, *p* = 0.0007), while 5-HIAA (*t* = −2.722, *p* = 0.001) and 5-HT turnover (*t* = −6.391, *p* < 0.0001) were higher. We also observed a lower 5-HT turnover in the CPu (*t* = 2.760, *p* = 0.001) but no change regarding 5-HT (*t* = −0.900, *p* = 0.38) or 5-HIAA levels (*t* = −1.055, *p* = 0.31). Regarding PrL/IL and Amy, ASD induced no change in 5-HT (PrL/IL: *t* = −0.803, *p* = 0.42; Amy: *t* = 0.096, *p* = 0.92), 5-HIAA (PrL/IL: *t* = −0.798, *p* = 0.43; Amy: *t* = −0.605, *p* = 0.55), and 5-HT turnover (PrL/IL: *t* = 0.872, *p* = 0.39; Amy: *U* = −1.162, *p* = 0.38, Table 1).

**Table 1.** Quantification of serotonin in brain areas involved during the MGT

	ASD	5-HT			5-HIAA			Ratio 5-HT	
		Control	*P* value	ASD	Control	*P* value	ASD	Control	*P* value
OFC	796 ± 55.7	1174 ± 85.7	*p = 0.01	576 ± 38.6	1006 ± 78.4	*p = 0.001	0.72 ± 0.02	0.85 ± 0.02	*p = 0.03
PrL/IL	717 ± 21.2	1583 ± 123.7	*p* = 0.42	1453 ± 40.5	667 ± 100.4	*p* = 0.43	2.03 ± 0.04	2.13 ± 0.02	*p* = 0.39
Cins	907 ± 28.5	800 ± 12.7	*p = 0.04	679 ± 31.0	609 ± 28.4	*p* = 0.28	0.74 ± 0.02	0.76 ± 0.02	*p* = 0.78
Amy	1359 ± 47.9	1369 ± 92.2	*p* = 0.92	923 ± 49.4	864 ± 43.2	*p* = 0.55	0.67 ± 0.02	0.63 ± 0.01	*p* = 0.38
Hippocampus	897 ± 38.7	1259 ± 78.9	*p = 0.0007	1536 ± 74.9	1134 ± 27.8	*p = 0.001	1.72 ± 0.06	0.90 ± 0.05	*p < 0.0001
CPu	1579 ± 61.2	1466 ± 90.3	*p* = 0.38	1072 ± 43.2	1169 ± 94.2	*p* = 0.31	0.68 ± 0.01	0.79 ± 0.06	*p = 0.001
									*p < 0.05

Serotonin (5-HT), 5-HIAA, and 5-HT turnover (5-HT/5-HIAA) levels (mean ± SEM) in the OFC, Pre and Infra limbic (PrL/IL), CIns, Amy, hippocampus, and CPu for control animals (*n* = 3) and sleep debt animals (ASD, *n* = 12, *p < 0.05). ASD animals exhibited lower 5-HT, 5-HIAA, and 5-HT turnover in the OFC; higher 5-HT level in the CIns, lower 5-HT level in the hippocampus but higher 5-HIAA and 5-HT turnover and lower 5-HT turnover in the CPu (*p <0.05 in bold).

As shown in Figure 6B, ASD animals exhibited a higher level of DA (*t* = −3.026, *p* = 0.01) and DOPAC (*t* = −2.363, *p* = 0.03) in the CPu, but no difference could be evidenced regarding HVA (*t* = −1.389, *p* = 0.19) and DA turnover (*t* = −1.442, *p* = 0.17). ASD induced higher DA level only in the CIns and CPu (OFC: *t* = −0.184, *p* = 0.85; PrL/IL: *t* = −1.528, *p* = 0.15; CIns: *t* = −2.964, *p* = 0.01; Amy: *U* = 0.084, *p* = 0.93; hippocampus: *t* = 0.552, *p* = 0.59; CPu: *t* = −3.026, *p* = 0.01, Table 2).

**Table 2.** Quantification of DA and NA in brain areas involved during the MGT

	ASD	DA			NA	
		Control	*P* value	ASD	Control	*P* value
OFC	1819 ± 53.7	1799 ± 82.3	*p* = 0.85	1409 ± 58.7	1499 ± 90.4	*p* = 0.46
PrL/IL	2302 ± 68.2	2089 ± 100.1	*p* = 0.15	1327 ± 38.9	1209 ± 71.8	*p* = 0.16
Cins	1993 ± 46.7	1711 ± 67.9	*p = 0.01	1282 ± 57	1129 ± 106.9	*p* = 0.22
Amy	9737 ± 402.2	9818 ± 1154.3	*p* = 0.93	7314 ± 409.8	6577 ± 37.6	*p* = 0.38
Hippocampus	1853 ± 45.5	1914 ± 177.1	*p* = 0.59	1915 ± 61	1873 ± 67.1	*p* = 0.74
CPu	72790 ± 2280.9	59082 ± 1434.9	*p = 0.01	4226 ± 205.3	384.4 ± 95.1	*p* = 0.39
						*p < 0.05

DA and NA levels (mean ± SEM) in the OFC, Pre and Infra limbic (PrL/IL), CIns, Amy, hippocampus, and CPu for control animals (*n* = 3) and sleep debt animals (ASD, *n* = 12, *p < 0.05). ASD animals also exhibited higher DA in the CIns and the CPu (*p <0.05 in bold).

NA levels did not change between control and ASD animals in the OFC (*t* = 0.749, *p* = 0.46), PrL/IL (*t* = −1.463, *p* = 0.16), CIns (*t* = −1.288, *p* = 0.22), Amy (*t* = −0.901, *p* = 0.38), hippocampus (*t* = −0.338, *p* = 0.74), or CPu (*t* = −0.983, *p* = 0.39, Table 2).

### Heterogeneous effects of ASD when applied before the exploitation phase

#### ASD applied before exploitation phase

**Figure 7.**
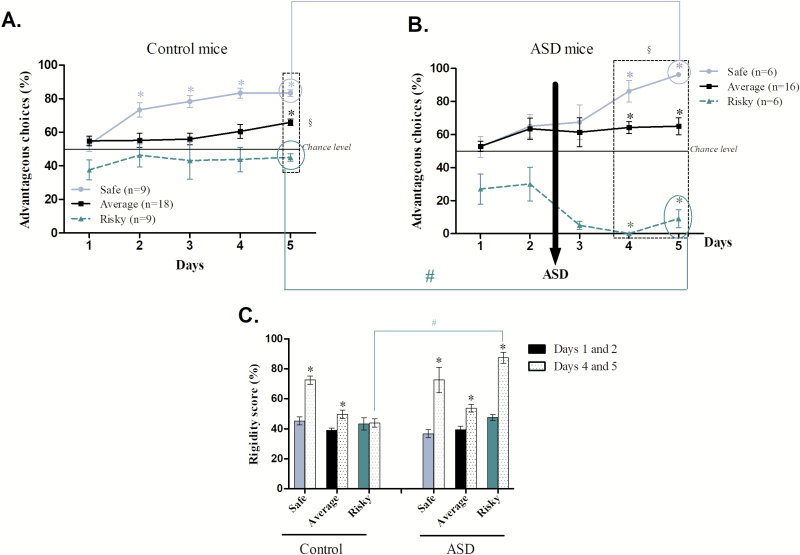
Development of mouse preferences during MGT regarding interindividual differences for control (A) and ASD mice (B): safe (blue), average (black), and risky (green dotted line) subgroups. Safe and Average mice preferences differed from day 1 (*p < 0.05), while risky control mice did not. ASD-risky mice chose significantly disadvantageous options (*p < 0.05). The three sub-groups of mice differed from each other during the last days (^§^*p* < 0.05). ASD-safe and ASD-risky mice differed from their control groups at the end of the MGT (^#^*p* < 0.05). C. Rigidity scores are reflected by the percentage of chosen arms during the 2 first days (filled) and the 2 last days (dotted), i.e. respectively, before and after ASD, for control and ASD mice. *Statistical difference between the first and the last 2 days of the task (*p* < 0.05). ^#^Statistical difference between control and ASD mice (*p* < 0.05).

As a group, control mice favored advantageous options at the end of the task, but interindividual differences emerged. Indeed, choices of 25% of animals did not differ from the first day of MGT (45 ± 6.3%), i.e. “risky” animals (Bonferroni correction, *p* < 0.012; day 1 *vs.* day 2: *t* = −2.530, *p* = 0.03; day 1 *vs.* day 3: *t* = −1.219, *p* = 0.25; day 1 *vs.* day 4: *t* = −1.000, *p* = 0.34; day 1 *vs.* day 5: *Z* = −1.521, *p* = 0.12). By contrast, animals from the “average” group (50%) developed a significant preference for advantageous options (Bonferroni correction, *p* < 0.012; day 1 *vs.* day 2: *t* = 0.271, *p* = 0.78; day 1 *vs.* day 3: *Z* = −0.388, *p* = 0.69; day 1 *vs.* day 4: *Z* = −0.305, *p* = 0.76; day 1 *vs.* day 5: *t* = 4.295, *p* = 0.0005). Animals from the group “safe” (25%) developed an even more marked preference for advantageous options (Bonferroni correction, *p* < 0.012; day 1 *vs.* day 2: *Z* = −2.266, *p* = 0.007; day 1 *vs.* day 3: *Z* = −2.488, *p* = 0.01; day 1 *vs.* day 4: *Z* = −2.666, *p* = 0.007; day 1 *vs.* day 5: *Z* = −2.666, *p* = 0.007; Figure 7A).

The same observation could be done for ASD mice (Figure 7B). Indeed, at the end of the MGT three groups of preferences emerged: “safe” (21%), “average” (78%) and “risky” mice (21%). “Safe” (Bonferroni correction, *p* < 0.012; day 1 *vs.* day 2: *t* = −1.775, *p* = 0.13; day 1 *vs.* day 3: *t* = −2.180, *p* = 0.08; day 1 *vs.* day 4: *t* = −4.886, *p* = 0.004; day 1 *vs.* day 5: *t* = −7.925, *p* = 0.0005) and “average” mice (Bonferroni correction, *p* < 0.012; day 1 *vs.* day 2: *t* = −1.649, *p* = 0.11; day 1 *vs.* day 3: *t* = −2.443, *p* = 0.02; day 1 *vs.* day 4: *t* = −4.697, *p* = 0.003; day 1 *vs.* day 5: *t* = −3.432, *p* = 0.003) preferred advantageous options at the end of the task compared to beginning of the task. However, ASD “risky” mice preferred disadvantageous options at the end of the task compared with the beginning (Bonferroni correction, *p* < 0.012; day 1 *vs.* day 2: *t* = −0.250, *p* = 0.81; day 1 *vs.* day 3: *t* = −2.026, *p* = 0.09; day 1 *vs.* day 4: *t* = 4.165, *p* = 0.008; day 1 *vs.* day 5: *t* = 2.000, *p* = 0.01). “Safe”, “average,” and “risky” groups differed from each other for the last two days (day 4: safe *vs.* average: *U* = 11.500, *p* = 0.007; safe *vs.* risky: *U′* = 36.000, *p* = 0.004; risky *vs.* average: *U′* = 96.000, *p* = 0.0004; day 5: safe *vs.* average: *U* = 1.000, *p* = 0.0005; safe *vs.* risky: *U′* = 36.000, *p* = 0.004; risky *vs.* average: *U′* = 96.000, *p* = 0.0004).

As shown in Figure 5C, these behavioral differences could not be explained by weight differences between subgroups (day 1: *H* = 1.555, *p* = 0.46; day 2: *H* = 3.117, *p* = 0.21; day 3: *H* = 3.723, *p* = 0.15; day 4: *H* = 2.171, *p* = 0.34; day 5: *H* = 0.522, *p* = 0.77). However, subgroup strategies led ASD risky mice to obtain less food pellets (day 1: *H* = 3.202, *p* = 0.20; day 2: *H* = 5.798, *p* = 0.05; day 3: *H* = 6.639, *p* = 0.04; day 4: *H* = 7.528, *p* = 0.02; day 5: *H* = 12.745, *p* = 0.002) and more quinine pellets during the three last days (day 1: *H* = 3.415, *p* = 0.18; day 2: *H* = 4.616, *p* = 0.09; day 3: *H* = 6.395, *p* = 0.04; day 4: *H* = 8.191, *p* = 0.02; day 5: *H* = 13.751, *p* = 0.001) compared with the ASD “safe” and “average” mice (Figure 5D).

Although ASD mice were like control mice distributed following a Gaussian distribution, their individual profiles were exacerbated with “safe” ASD mice choosing significantly more advantageous options than “safe” control mice (*U* = −2.339, *p* = 0.03) and “risky” ASD mice choosing significantly and clearly less advantageous options than “risky” control mice (*U* = −3.182, *p* = 0.001). Conversely, ASD did not change “average” mouse preferences (*t* = 0.766, *p* = 0.44), leaving this subgroup relatively unaffected by ASD (Figure 7).

Rigidity score is expected to change over days because mice first explored all options and then chose to exploit preferentially some of them. Control “safe” mice chose the same arm 45% of the time at the beginning of MGT and the same arm 72% of the time at the end of the task (*t* = −6.810, *p* = 0.003; Figure 7C). Likewise, “average” control mice chose the same arm 39% of the time at the beginning of the MGT and 50% of the time the same arm at the end of the task (*Z* = −2.755, *p* = 0.006). Conversely, control “risky” mice continued to explore for the entire duration of the experiment (*t* = −0.133, *p* = 0.89). Compared with their control subgroups, we observed exactly the same changes of the rigidity score for ASD “safe” (*t* = −3.616, *p* = 0.01) and “average” mice (*t* = −4.105, *p* = 0.0009). On the contrary, ASD “risky” mice displayed a significant increase of their rigidity score over days (*t* = −7.746, *p* = 0.0006), thus drastically drifting apart from control “risky” mice (*t* = −9.869, *p* < 0.0001).

In summary, ASD exacerbated “safe” and “risky” behavioral profiles through an amplification of their natural preference and the rigidity scores of “risky” mice and left “average” mice largely unaffected.

#### Prediction of sleep vulnerability

We defined as “vulnerable” the mice that had a mean of preference during day 1 and day 2 lower than 40%. We observed that ASD applied before the exploitation phase had a significant more important effect on these “vulnerable” mice than on the other mice. Indeed, ASD applied at this time led to 22% of decrease of their preference for advantageous choices (*t* = −2.202, *p* = 0.04). However, ASD applied after the exploitation phase did not have any important effect on “vulnerable” mice (*t* = 0.522, *p* = 0.60).

In others words, we observed a significant effect on mice that initially showed a percentage of advantageous choices inferior to 40% only when ASD was applied at a specific moment of the decision-making task. Indeed, mice with a percentage of advantageous choices inferior to 40% during day 1 and day 2 but which were not then under ASD or which were under ASD after the exploitation phase did not show any interaction between ASD, Vulnerable, and Time. We were thus able to predict mice vulnerability to sleep debt by distinguishing animals before ASD and observing the effect of ASD on mice depending on this previous selection.

We also observed that ASD had a more important effect on the rigidity of these “vulnerable” mice as it increased by 30% their rigidity score at the end of the task (*t* = 2.491, *p* = 0.01).

In summary, ASD increased heterogeneity in mice preference at the end of the MGT and had a more important effect on decision-making strategies of “vulnerable” mice, defined before ASD, only when applied before the exploitation phase.

## Discussion

In contrast to the well-known benefits of sleep for the body and the brain [41], knowledge about cognitive deficits due to sleep loss are lacking [10]. Behavioral deficits related to an ASD were mostly conducted on humans. Studies using animals (mostly rodents) have mainly shown that ASD impairs memory consolidation, vigilance, sustained attention, and to a lesser extent, executive processes [34, 42, 43]. To our knowledge, no studies have documented ASD effect on decision-making in rodent. We studied the effect of ASD at two moments of the decision-making processes. We showed that whenever ASD was applied it disturbed decision-making processes. However, we observed a tendency to make more advantageous choices at the end of the MGT when ASD was applied after the establishment of individual preference (after the exploitation phase). We thus hypothesized that the moment of application of ASD during decision-making processes is important: ASD has more deleterious effect if applied before the establishment of individual preference (before the exploitation phase). Even though mice could sleep for 2 more days after ASD applied before the exploitation phase, their decision-making processes were no more efficient. This observation suggested that ASD effects could not be explained by global memory deficits, but rather by preventing the selection of advantageous options at a key step of the decision-making process. Besides, and more importantly, these ASD-related behavioral modifications were associated with a decrease of cognitive flexibility. These results match previous ones showing that ASD is responsible for perseverative behaviors in humans [6, 8, 9]. Our control experiments ruled out the effect of stress related to ASD, as plasma corticosterone measures did not show any difference between control and ASD mice. They also excluded modification of anxiety, of reward sensitivity or locomotor activity related to ASD. Whenever ASD was applied it was associated with a decrease of choice latency during MGT. These results are in accordance with those of Berro and colleagues who have shown that 6 hours of continuous wakefulness induced impulsive-like behavior in cocaine-treated mice. Since these authors admitted that such ASD (a slightly one compared with our 23 hours of continuous wakefulness) is responsible for a potentiation of impulsivity of mice under cocaine effect, it would be reasonable to suggest that our ASD would be responsible for an impulsive behavior in mice *per se* [44]. Therefore, we can reasonably think that ASD, applied at a key step, disrupts decision-making process by preventing mice from developing efficient strategies through a decrease in their cognitive flexibility and an increase of their impulsivity.

To better understand on which level ASD impair decision-making, we measured the amount of NA, DA, 5-HT, at the end of the task in different brain areas, which we previously showed to be implicated in decision-making [31]. We also measured the 5-HT turnover in all brain areas and the DA turnover in the CPu. We showed that ASD applied at a key step of decision-making process powerfully, even after 2 days of sleep, disturbs brain neurochemistry with a decrease of 5-HT amount in OFC and an increase of DA amount in the dorsal striatum, a neurochemical imbalance that has been associated with significant cognitive rigidity in monkeys [45]. It has also been demonstrated in the marmoset that prefrontal 5-HT depletion (in particular in the OFC) was sufficient to produce cognitive rigidity with persevering responses [12]. ASD is also responsible for significant reduction of metabolic activity in several human brain regions (e.g. frontal cortex, thalamus) with only a partial restorative effect on metabolic activity in frontal areas after one night of sleep recovery [46]. Our results are in accordance with these data since, 2 days after ASD, the 5-HT level and turnover remained decreased in the OFC. We could thus hypothesize that ASD would be responsible for immediate and long-lasting decreases of 5-HT activity in specific prefrontal subareas such as the OFC, which would indirectly induce cognitive inflexibility and maladapted decision-making strategies. Catechol-O-methyl-transferase (COMT) catalyzes DA and DOPAC into HVA, while monoamine-oxidase catalyzes DA into DOPAC and HVA [47]. We may hypothesize that ASD increases amount of DA in the dorsal striatum *via* a reduction of COMT activity as we observed an increase of DOPAC with no change of neither HVA level nor DA turnover. However, this hypothesis remains to be fully investigated. Nevertheless, it has been shown that dorsal medial striatum is critical for both learning and expression of goal-directed behavior possibly involving DA neurotransmission [48]. Enhanced dopaminergic tone in the dorsal striatum results in hyperactivity and dopaminergic transmission in the dorsal striatum mediates habit formation [49]. Our results also showed that the 5-HT turnover (ratio 5-HIAA/5-HT) in the CPu, was decreased 2 days after ASD. It has been hypothesized that in dorsal striatum 5-HT release might adjust the signal-to-noise ratio of the striatal network to improve action-selection performance [50]. Therefore, low 5-HT levels and turnover in OFC combined with low 5-HT turnover and high level of DA and DOPAC (potentially via low COMT activity) in the CPu, observed even 2 days after ASD, could be the neurobiological substrates by which ASD promotes impulsivity and cognitive rigidity, and consequently impacts decision-making efficiency through animal’s difficulties to choose long-term advantageous options during the MGT.

We also show that DA amounts were increased, 2 days after ASD, in CIns without any change in the OFC, Pre and Infra limbic cortices (PrL/IL), Amy, and hippocampus. Moreover, 5-HT levels were higher in the CIns 2 days after ASD. The role of DA in the CIns is not clearly defined, but the 5-HT system in the CIns has been linked with disgust reactions [51–53]. We could thus hypothesize that ASD leads to a higher level of 5-HT in the CIns that will alter disgust feelings for quinine pellets during the MGT. We finally observed a low level of 5-HT and a high 5-HT turnover in the hippocampus. Reports in the literature concerning ASD and 5-HT in the hippocampus are quite unclear. Indeed, 5-HT amounts were found to increase [54, 55] or to decrease [56] during ASD. Regarding the 5-HIAA/5-HT turnover, Asikainen and colleagues (1995) found that, following 4 hours of continuous wakefulness, it was significantly increased in the hippocampus [57], which is in accordance with our results. This would suggest that following ASD, the 5-HT turnover increase in the hippocampus and thus indirectly has an impact on the 5-HT amount.

Our previous results have shown that spontaneous individual profiles emerged in control mice regarding MGT choices: “safe”, “average,” and “risky” profiles. Indeed, the majority of control mice (50%, “average”) preferred advantageous options without neglecting alternative—potentially riskier—choices. The “safe” ones (25%) were guided by risk avoidance and were more affected by their internal state [31]. The “risky” ones (25%) were more attracted by environmental cues and maintained high level of exploration, including that of risky options [30, 31]. A majority of ASD-mice (58%) had exactly the same profile as control “average” ones. However, after ASD we observed that “risky”-ASD mice (21%) strongly preferred the disadvantageous choices from the third day until the end of the task. The “safe”-ASD mice (21%) preferred significantly more long-term advantageous choices or similarly were more guided by risk avoidance compared with control “safe” ones. The fact that “risky” mice preference changed drastically in favor of disadvantageous options, with both a marked decrease of pellet consumption and an increase of quinine pellet obtained, is in accordance with human studies showing increased risk-taking behaviors in sleep-deprived subjects [6] with blindness for future loss [58]. Our “risky”-ASD mice maintained a disturbed behavioral pattern reminiscent that of patients with ventromedial prefrontal lesion [59], a brain area that contains OFC and exhibits reduced metabolism even after one night of sleep recovery following ASD as compared with other brain areas [46]. Finally, this “risky”-ASD mice were the only ones to be strongly affected by ASD with a large increase of cognitive rigidity (30%), which could be linked to both a strong desire to get a large reward immediately and/or be associated to less sensitivity to penalty leading to risk-taking proneness.

Here we also looked at the heterogeneity of animal choices after ASD and we observed that it was larger after ASD and this observation was even more obvious if ASD was performed before the exploitation phase than after. We therefore hypothesized that ASD increased variability of preference at the end of the MGT leading to a lack of improvement of advantageous choices as a group. We then used the linear regression analysis to question the possibility to predict which mice would be the most vulnerable to ASD depending of their behavior before ASD and its moment of application. We found that ASD has a drastic effect only in mice with initially risky behavior and if ASD was applied before the exploitation phase. These results are very interesting because we were able for the first time to predict an individual cognitive vulnerability related to sleep debt in mice. Moreover, these results are in accordance with human studies showing that individual differences already existing are amplified by ASD [23, 24, 27, 28].

Altogether, these results suggest that ASD altered decision making processes and that this observation is mostly due to an increase of heterogeneity between mice that are more or less vulnerable to sleep debt. Moreover, ASD-mediated disruption of the balance between 5-HT and DA within prefronto-striatal networks could provoke a specific decrease of behavioral flexibility and the development of nonoptimal decision-making strategies. Besides, we showed that ASD applied before the exploitation phase has a more important effect on 21% of mice. Therefore, we evidenced for the first time a cognitive vulnerability to ASD in a healthy inbred mouse population and were able to predict this vulnerability for 83% of them (18% of the total group of mice).
